# Insights from the Applications of Single-Cell Transcriptomic Analysis in Germ Cell Development and Reproductive Medicine

**DOI:** 10.3390/ijms22020823

**Published:** 2021-01-15

**Authors:** Hyeonwoo La, Hyunjin Yoo, Eun Joo Lee, Nguyen Xuan Thang, Hee Jin Choi, Jeongheon Oh, Ji Hyun Park, Kwonho Hong

**Affiliations:** Department of Stem Cell and Regenerative Biotechnology, Institute of Advanced Regenerative Science, Konkuk University, Seoul 05029, Korea; lahw94@gmail.com (H.L.); hyunjinyoo7@gmail.com (H.Y.); ejlee1824@gmail.com (E.J.L.); thang.nx1012@gmail.com (N.X.T.); heejinchoi1123@gmail.com (H.J.C.); jeongheon33@gmail.com (J.O.); jihyunpark0522@gmail.com (J.H.P.)

**Keywords:** scRNA-seq, germ cell, transcriptome, reproductive medicine, fertility

## Abstract

Mechanistic understanding of germ cell formation at a genome-scale level can aid in developing novel therapeutic strategies for infertility. Germ cell formation is a complex process that is regulated by various mechanisms, including epigenetic regulation, germ cell-specific gene transcription, and meiosis. Gonads contain a limited number of germ cells at various stages of differentiation. Hence, genome-scale analysis of germ cells at the single-cell level is challenging. Conventional genome-scale approaches cannot delineate the landscape of genomic, transcriptomic, and epigenomic diversity or heterogeneity in the differentiating germ cells of gonads. Recent advances in single-cell genomic techniques along with single-cell isolation methods, such as microfluidics and fluorescence-activated cell sorting, have helped elucidate the mechanisms underlying germ cell development and reproductive disorders in humans. In this review, the history of single-cell transcriptomic analysis and their technical advantages over the conventional methods have been discussed. Additionally, recent applications of single-cell transcriptomic analysis for analyzing germ cells have been summarized.

## 1. Introduction

The male and female germ cells combine to form the zygote, and this process is called fertilization. The development of fertilization-competent germ cells involves complex regulatory processes, including germ cell-specific cell division (meiosis), re-establishment of sex-specific imprinting genes, and acquisition of sex-specific dimorphic characteristics [1,2,3]. Various studies have attempted to elucidate the mechanism underlying germ cell development using several model systems. The key biological pathways and molecules involved in germ cell development and fertilization have been identified. In the field of reproductive medicine, these molecules serve as diagnostic and therapeutic biomarkers for patients with reproductive disorders [4,5].

Genome-scale analyses of germ cells provide promising insights into the fields of developmental biology and reproductive medicine. However, the numbers of developing and meiotic germ cells are limited. Hence, conventional genome analysis approaches have limitations to delineate genomic, transcriptomic, and epigenomic regulation at a single-cell resolution. In the conventional bulk sequencing method, numerous heterogeneous cells are subjected to sequencing. Most studies have adopted the bulk sequencing method, which can capture global or representative gene expression patterns or chromatin conformations of the pooled cells. However, this method does not account for cell-to-cell heterogeneity. The differentiation of immature germ cells, including progenitor primordial germ cells (pre-PGCs) and primordial germ cells (PGCs), into mature germ cells involves various steps [1,6]. Thus, a small degree of epigenomic heterogeneity could result in distant cell fate, which is not captured by bulk sequencing. To overcome this limitation, single-cell sequencing (SC-seq) was developed in the last decade [7]. The SC-seq can identify the developmental fate of each cell. The SC-seq technique was first developed using germ cells (oocytes) and preimplantation embryos (blastocysts). Various studies have improved the single-cell isolation and sequencing library preparation techniques. Currently, the most common method of SC-seq is single-cell RNA sequencing (scRNA-seq). The scRNA-seq can identify cell-to-cell heterogeneity within a mixed cell population without averaging the cell-specific gene expression levels. Additionally, scRNA-seq enables cell lineage tracing analysis. Cell heterogeneity from the scRNA-seq data can be visualized using principal component analysis, t-stochastic neighbor embedding (t-SNE), or uniform manifold approximation and projection [8,9]. The plots display cells with similar sequencing read characteristics as a cluster. The analysis of a sufficient number of cells can reveal their lineage trajectory, which could provide valuable information for low-input and complex samples. The scRNA-seq can be a useful tool to analyze rare and scarce target cells. Bulk sequencing involves cell sorting techniques, such as fluorescence-activated cell sorting (FACS) and magnetic-activated cell sorting (MACS), to isolate the target cells. However, the low number of rare and mixed cell types is a major limitation for sorting these cells as they yield a small library size for bulk sequencing. If the rare cells are not impaired during sequencing, scRNA-seq can bypass the cell sorting and isolation procedures and capture their unique characteristics. Therefore, scRNA-seq can be employed in studies involving germ cells, zygotes, and preimplantation embryos. In this review, we discuss recent progress in germ cell studies that have employed single-cell genomic technology.

## 2. Evolution of scRNA-seq Technique 

**Figure 1:**
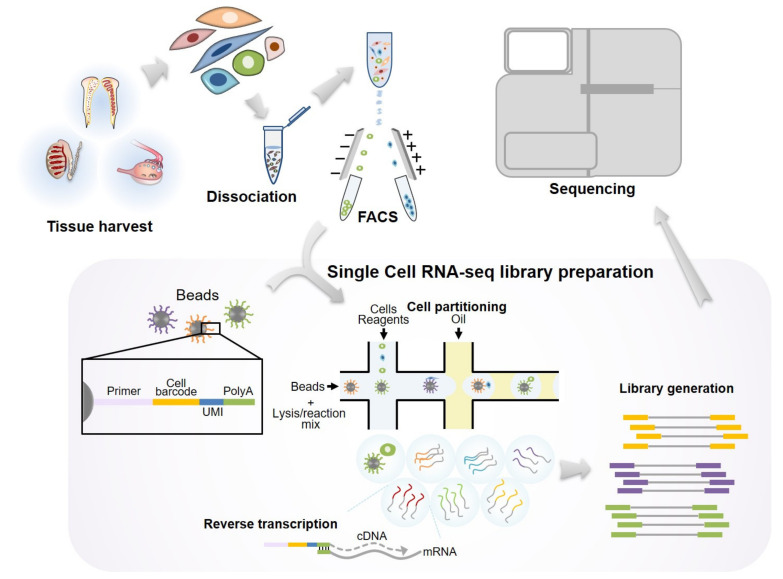
Schematic illustration showing the procedure of scRNA-seq in gonadal tissues. Reproductive tissues are isolated and enzymatically dissociated. Highly pure single cell populations are obtained by conventional cell sorting methods such as fluorescence-activated cell sorting (FACS) or magnetic-activated cell sorting (MACS). Uniquely barcoded beads are required for microfluid-based scRNA-seq. Technically, one cell is interacted with a bead, and subsequently the cells are subjected to cell lysis for the preparation of mRNAs. The isolated mRNAs are used for reverse transcription. Finally, scRNA-seq libraries containing bead-specific oligo sequences and unique molecular identifier (UMI) are generated.

The scRNA-seq was first used to examine the transcriptome of mouse oocytes and blastocysts and identify the aberrantly expressed genes in *Dicer1* or *Ago2* knockout oocytes and blastocysts [7]. The study reported that scRNA-seq identified a higher number of differentially expressed genes (DEGs) than microarray analysis. Other studies have modified and improved the scRNA-seq protocol. The advanced methods include Smart-seq [10,11], CEL-seq [12,13], Qualtz-seq [14], MARS-seq [15], Cyto-seq [16], SUPeR-seq [17], Drop-seq [18], InDrop [19], MATQ-seq [20], Chromium [21], sci-RNA-seq [22], Seq-Well [23], DroNC-seq [24], and SPLiT-seq [25] (Table 1). Generally, scRNA-seq involves the following steps: preparation of in vitro or in vivo samples, dissociation of the sample into single cells, barcode tagmentation of individual cells and reverse transcription, library preparation, massively parallel sequencing, and downstream bioinformatics analysis (Figure 1). Various scRNA-seq methods differ in at least one of the aforementioned steps. Furthermore, some scRNA-seq protocols, including Drop-seq [18], InDrop [19], and Chromium [21], utilize droplet-based technologies in which dissociated individual cells are encapsulated into oil droplets and subjected to barcode tagmentation as well as amplification using microfluidic devices [26]. These methods are suitable for analyzing samples containing mixed cell populations, examining transcriptomic heterogeneity in the mixed cell population, and cell lineage tracing experiments. When Tang et al. first introduced scRNA-seq [7], the method did not involve microfluidic manipulation as individual oocytes or preimplantation embryos were manually selected under the microscope. In addition to the manual single-cell isolation methods, the conventional cell separation techniques, including FACS, MACS, and laser capture microdissection, have been employed for single-cell separation and harvesting. The sequencing read coverage also varies among the scRNA-seq methods. Smart-seq [10], MATQ-seq [20], and SUPeR-seq [17] can sequence almost full-length transcripts, whereas other methods can sequence either 5′ end (STRT-seq) or 3′ end (Drop-seq [18], DroNC-seq [24], Seq-Well [23], and SPLiT-seq [25]) of the transcripts. The full-length sequencing method, which can detect splice variants and strand-specific transcripts, has more advantages than the methods that sequence 5′ or 3′ ends of the transcripts. MATQ-seq [20] and SUPeR-seq [17], which are reported to detect both polyA(+) and polyA(−) transcripts simultaneously, are optimized for the examination of non-coding RNAs.

The signal-to-noise ratio of scRNA-seq is low owing to the low amount of input sequences. To overcome this limitation, a normalization method for measuring endogenous transcript levels should be employed. Currently, unique molecular identifiers (UMIs) or spike-in controls have been used for normalization [27]. The UMIs are used to determine the absolute transcript levels. Spike-ins, such as the external RNA control consortium controls from different species with known sequences and concentrations, are used to calculate the relative levels of endogenous transcripts. Previous studies have demonstrated that UMIs (approximately 5 bp in length) can reduce technical noise and aid in fitting the sequencing reads into statistical models [28,29,30]. Spike-in controls with known concentrations of synthetic transcripts can be used to calculate the differences between expected and observed expression of the spike-ins along with a cell type-specific factor that adjusts the difference. Next, the cell type-specific factor is applied to obtain the normalized level of endogenous transcripts. The spike-in normalization method has been successfully used in the development of statistical models that can be applied to various scRNA-seq experiments [31,32,33].

## 3. Germ Cell Development

**Figure 2:**
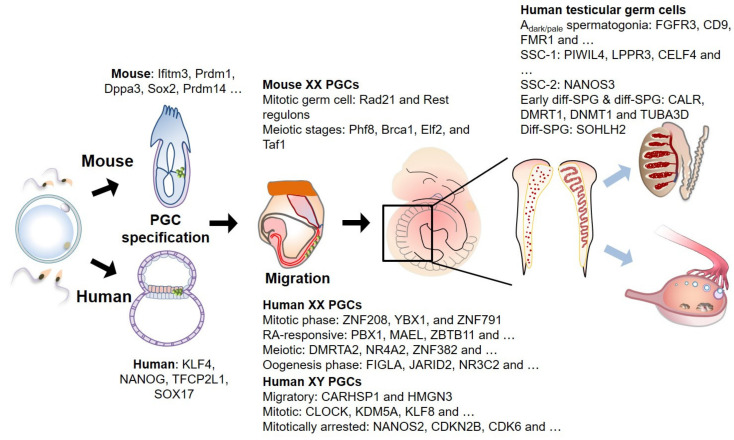
Human and mouse germ cell development and associated genes. Primordial germ cells (PGCs, marked as green) can be recognized for the first time at the extraembryonic region of epiblast in mouse (at ~E6.25) and a layer between epiblast and visceral endoderm in human (at ~2 to 3 weeks of gestation) during gastrulation. These cells migrate towards the genital ridge during embryo turning, and simultaneously undergo extensive epigenetic reprogramming. Upon arrival at the genital ridge, PGCs are dispersed in the female genital ridge and organized to make a winding tubular pattern in male genital ridge. Multiple scRNA-seq studies in various stages of germ cell development were performed to elucidate cellular diversity, and critical gene expression signatures in developing germ cells, terminating mitosis and entering meiosis. Stage-specific genes identified by scRNA-seq are noted. SSC: spermatogenic stem cells, diff-SPG: differentiating spermatogonium.

Mouse and human germ cells are unipotent cells that can differentiate into oocytes or sperms [1,34,35]. In mice, the germ cells begin to form a subset of specialized mesoderm-origin cells called PGCs at the extraembryonic region of the epiblast during gastrulation (Figure 2). The specified PGCs then migrate and colonize the genital ridge. The migrating PGCs are reported to undergo epigenetic reprogramming, including global DNA demethylation, imprinting erasure and re-establishment, and histone methylation (H3K9me2 and H3K27me3) [36,37,38]. The bone morphogenetic protein (BMP)- small mother against decapentaplegic (SMAD) signaling axis mediates PGC specification by activating critical transcription factors (TFs), including BLIMP1, PRDM14, and TFAP2C [39,40] (Figure 2). The TF-regulated transcriptional circuit modulates the activation of germ cell-specific gene expression and repression of somatic cell lineage-specific gene expression [41,42,43,44]. The loss of at least one of the key TFs leads to impaired PGC specification and repression of mature germ cell formation.

The male and female germ cells undergo dimorphic differentiation processes after they reach the genital ridge [45]. In the genital ridge, the male germ cells become mitotically quiescent (arrested at G0/G1 phase) after several cell divisions and begin to proliferate after birth [46]. The proliferating male germ cells colonize at the base of the seminiferous tubule and transform into spermatogonial stem cells, which are diploid cells that give rise to mature spermatozoa [47]. In contrast, the female PGCs reach the genital ridge and undergo meiosis I. The cell cycle of female PGCs is arrested at the diplotene of meiotic prophase I. During puberty, the female germ cells resume meiosis I, enter meiosis II, and complete meiosis II after fertilization [48].

Various studies have demonstrated that transcriptional regulation by TFs is conserved using an embryonic stem cell (ESC)-derived in vitro germ cell differentiation model. However, the downstream gene networks in humans are distinct from those in mice. For example, a group of pluripotent genes, comprising *Sox2*, *Esrrb*, and *Klf2*, are expressed in mouse PGCs, whereas *KLF4* and *TFCP2L1* are expressed in human PGC (hPGC)-like cells (Figure 2). SOX17 upregulates the expression of BLIMP1 and TFAP2C in hPGCs, which is not observed in mouse PGCs. The formation of PGC-like cells from ESCs is hindered upon the loss of SOX17 [34]. Therefore, these studies suggest the presence of both common and unique TF circuits during PGC development across different species.

## 4. Findings from scRNA-seq Studies in PGCs

Yabuta et al. demonstrated that Ifitm3, Prdm1, Dppa3, Sox2, Prdm14, Nanos3, Kit, and Dnd were exclusively expressed in PGCs in at least one of the E6.75–E8.25 stages during early mouse PGC specification. *Sox2* and *Prdm1* were specifically expressed in PGCs. The expression of *Sox17* and *Sox3* was transiently upregulated at E7.25. In contrast, the expression of *Prdm14*, *Nanos3*, *Kit*, and *Dppa3* was upregulated after E7.25 [49]. In female PGCs, the expression levels of genes involved in mitosis and meiosis were significantly altered from E12.5 to E16.5. In particular, the expression of TFs, such as Rest and Trp53, was mainly detected in PGCs and oogonia. The expression of TFs associated with meiosis initiation, including Msx1, Msx2, Cdx2, Sox4, Gata2, and Bmyc, was markedly upregulated at the pre-leptotene stage of PGCs. Meanwhile, Dmrtc2 and Taf4b expression was upregulated in the late meiotic stage, whereas Taf9b expression was upregulated at the late meiotic stage of PGCs [50]. The expression of pluripotent genes, *Nanog* and *Oct4*, and their associated genes, *Dppa2* and *Dppa4*, was downregulated in PGCs at E11.5 [51]. Moreover, the expression patterns of cell cycle regulators (Ccna2, Ccnb1, Ccnd3, E2f1, E2f2, E2f3, E2f7, and E2f8) were similar to those of the pluripotent genes in PGCs. The differential transcriptomic profiles between XX and XY PGCs were observed even at E11.5. These genes were termed as chaperone complex-associated and proteasome/proteolysis-associated genes. The number of DEGs increased at later developmental stages. Moreover, RNA splicing may play an important role in the specification of XY PGCs. Nodal/Activin and BMP target genes are specifically expressed in E11.5 XX and XX PGCs, respectively.

In humans, PGC specification may begin approximately at the end of the third week in the layer between the epiblast and visceral endoderm [3] (Figure 2). In 2015, Guo et al., for the first time, performed scRNA-seq analysis on intact 4- to 19-week-old hPGCs. The authors reported that both male and female PGCs exhibited downregulated expression levels of pluripotency-associated genes after 4 weeks. The expression of meiosis-related genes was upregulated in 11- to 17-week-old female PGCs. Moreover, the reactivation of the inactivated X chromosome in female PGCs was observed after four weeks [52].

Chen et al. aimed to identify early hPGC progenitors from peri-implantation embryo cells using the ESC differentiation into PGC-like cell (hPGCLC) model. The hPGCs were the only type of human cells that simultaneously expressed NANOG, SOX17, and TFAP2C on day 12 of embryonic development [53]. The scRNA-seq analysis revealed a previously unnamed transitional state of pluripotency of cells upstream of hPGCLC during the germ cell differentiation process, which was termed as “germinal pluripotency.” Germinal pluripotency refers to a state in which the characteristics of both preimplantation epiblast naïve pluripotency and post-implantation embryo primed pluripotency are observed. The cell population can develop into hPGC or somatic cells with amnionic, gastrulating trophoblast, or extraembryonic mesenchymal cell characteristics. In this state, the pattern of gene expression is correlated with both preimplantation epiblast (naïve) and post-implantation late epiblast (primed), in which the cells exclusively and simultaneously express TFAP2A, TFAP2C, and SOX17. The analysis of TFs revealed that SOX17 specifies hPGCs and that TFAP2C allows TFAP2A-positive progenitors to bypass the Weismann’s barrier, which enables the maintenance of progenitor cell potency [54].

## 5. Findings from scRNA-seq Studies on Gonadal Germ Cells 

Guo et al. examined the later stages of hPGC development using scRNA-seq. Variations were observed in the transcriptome profiles of 4-week-old to 18-week-old embryos. The transcriptome profiles of hPGCs before 11 weeks, which migrate from the yolk sac endodermal layer to the genital ridge, did not vary. However, the transcriptome profile of individual PGCs varied after migrating to the genital ridge and meiosis initiation, whereas that of male PGCs did not markedly vary. The PGCs undergo multiple steps of development or extensive apoptosis after migrating into the genital ridge [55]. The number of human oogonia increases up to 7 million within 20–24 weeks of gestation and decreases to 2 million during parturition. 

The same research group also performed scRNA-seq analysis of human gonadal niche cells and fetal germ cells from 29 embryos aged 4–26 weeks. A subset of female or male fetal germ cells exhibited characteristic transcriptional and metabolic programs that were modulated by master TFs [56]. The analysis of sequencing data from all cells revealed four subpopulations of female fetal germ cells. Among them, the authors traced a cluster of fetal germ cells at the mitotic stage from 11-week-old embryos and the older embryos. This cell cluster is speculated to be involved in population maintenance. Another cluster of fetal germ cells, which started appearing at week 11 of gestation, was responsive to retinoic acid (RA) signaling. Additionally, this cluster may initiate meiosis. The meiotic prophase cluster was identified from 14-week-old embryos, while the oogenesis stage cluster was identified from 18-week-old and older embryos. The authors suggested that this was consistent with the results of previous studies that reported the presence of four distinct cell populations [57,58,59]. The scRNA-seq can distinguish the fetal germ cells from simultaneously sequenced non-germinal gonadal niche cells. Hence, scRNA-seq can identify the cell type-specific expression patterns and interactions. The study reported that BMP2 from female gonadal somatic cells activated the BMP signaling pathway in fetal germ cells and that DLL3 from fetal germ cells activated the NOTCH signaling pathway in gonadal somatic cells.

The TFs, such as ZNF208, YBX1, and ZNF791, are critical regulators of the mitotic phase in female PGCs. Additionally, the expression of genes in RA-responsive PGCs is regulated by TFs, such as PBX1, MAEL, ZBTB11, ZNF362, ZGLP1, HOXA5, HMGB3, HOXB6, and HES6. Furthermore, DMRTA2, NR4A2, ZNF382, PAXBP1, RLF, L3MBTL1, MGA, HSF2, LHX8, and ZIC4 regulate the meiotic cycle during PGC development. FIGLA, JARID2, NR3C2, AFF1, STAT1, NFKB2, and TBX3 seem to activate the transcriptional network in female gonads.

Male PGCs have been demonstrated to cluster into migrating, mitotic gonadal, and mitosis-arrested PGCs. The expression of some cell cycle arrest-related genes, including *NANOS2*, *CDKN2B*, and *CDK6*, was upregulated in mitosis-arrested male PGCs. POU5F1(+)/DDX4(−) PGCs were predominantly observed in male gonadal PGCs but not in female gonadal PGCs. In 18-week-old testes, POU5F1(+) PGCs were homogeneously detected in the seminiferous cords, whereas DDX4(+) PGCs were mainly detected in the peripheral zones. CARHSP1 and HMGN3 regulated the gene expression network in migratory male PGCs. CLOCK, KDM5A, KLF8, NKRF, MAEL, TEF, ZBTB43, MEF2D, SIX2, and SOX15 might be the critical TFs in mitotic PGCs. Similarly, DMRTB1, EBF3, FEZF1, MAEL, SATB2, SOX12, ZNF267, and ZSCAN5A might play important roles in mitosis-arrested PGCs.

## 6. TFs for Sex Determination 

The determination of PGC fate is dependent on signals from the surrounding cells rather than the innate genotype of the undifferentiated germ cell [60,61,62]. For example, XY PGCs develop into gonocyte-like germ cells when co-cultured with ovarian niche cells, whereas they develop into spermatocyte-like germ cells when co-cultured with testis cells. To elucidate the mechanism that regulates cellular processes, such as cell pluripotency and initiation or inhibition of meiosis, various studies have examined epigenomic dynamics throughout the prenatal development of germ cells.

One study subjected the cell populations in female mouse gonads to scRNA-seq to elucidate the molecular mechanism underlying female germ cell development [63]. The genital ridges of the E11.5 mouse embryo and the ovaries of the E12.5, E13.5, and E14.5 mouse embryos were analyzed. The sequencing read qualities of 19,387 cells were not sufficient. The analysis of known germ cell markers identified 13 clusters. Three clusters were annotated as germ cell clusters, while ten were annotated as non-germinal somatic cell clusters.

A t-SNE analysis revealed four different meiotic stages (meiotic stages I, II, III, and IV) and mitotic PGCs based on gene expression signatures and clustering. The number of germ cells at meiotic stage I significantly increased at E13.5, whereas that at meiotic stages II, III, and IV germ cells significantly increased at E14.5. The sets of genes were grouped into units of regulons based on co-expression modules and cis-regulatory motifs, and each cell was scored based on the activity of the regulons. The analysis revealed that mitotic germ cells and meiotic I germ cells expressed Sox2, Etv4, and Nanog regulons. The cell cycle regulons, including Rad21 and Rest, were expressed in mitotic germ cells, and the expression of these regulons was downregulated in the meiotic I germ cell cluster. The germ cells at meiotic stages II, III, and IV expressed Phf8, Brca1, Elf2, and Taf1. The expression of regulons, such as Kdm5a, Nr3c1, and Stat3, was gradually upregulated in the germ cells at meiotic stage IV.

Another study performed scRNA-seq in both male and female PGCs with a special focus on sex determination [51]. To represent the entire process of mouse PGC sex determination, whole gonads from E10.5 to E16.5 embryos were subjected to scRNA-seq. The cells expressing germ cell markers were selected for further analysis to identify the potential genes associated with germ cell development. The regulons were identified and their activity in each cell was scored. Next, the cells were classified based on sex and the order of pseudotime. That is, each sequenced cell is given a score based on its expression level of development-related genes and lined up in a putative order from the least differentiated one to the most differentiated one. The analysis identified STRA8 as a key regulator. Previous studies have reported that STRA8 expression is downregulated in developing mouse testes and that it is critically involved in female germ cell meiosis. RAD21 and YBX1 are two TFs that target *Stra8*. The expression patterns of Rad21 and Ybx1 initially were not specific to sex in the developing germ cells. However, the expression levels of Rad21 and Ybx1 were downregulated in the developing female germ cells during meiosis. This suggested that these TFs downregulated Stra8 expression. In contrast, KDM5A and PBX3 are putative positive regulators of Stra8 expression. KDM5A and PBX3 target the expression of *Stra8*. The expression of KDM5A and PBX3 is activated within a short period specifically in the developing female germ cells. Additionally, KDM5A negatively regulates the expression of *Rad21* and *Ybx1*. These findings indicate the presence of a regulatory network involving these genes that regulates Stra8 expression and promotes meiosis in developing germ cells. The transcriptome profiles of PGCs in the adrenal, ovarian, and testicular environments were comparatively analyzed. The marker gene expression patterns were similar among the ovarian and adrenal germ cells. In contrast to the ovarian germ cells, the adrenal germ cells did not exhibit the expression of Axin2, Lef1, and Sp5, which are WNT/β-catenin signaling pathway-related genes; Msx1 and Msx2, which are TFs; Cdx2, which is a cell cycle protein; and Figla, which is an oocyte-specific basic helix-loop-helix TF. The expression of most master regulator genes in testis germ cells was downregulated in the adrenal environment, which explained the lack of functionality of adrenal germ cells. These results demonstrated that scRNA-seq could elucidate transcriptomic regulation during early germ cell developmental processes.

## 7. scRNA-seq Studies on the Adult Ovary

The periodic menstrual cycle is one of the most dynamic physiological changes in humans. The menstrual cycle in humans is initiated from puberty and continues consecutively until menopause, unless interrupted by pregnancy or some endocrinal disorders. Additionally, the menstrual cycle is a major factor that regulates oocyte development [64]. During human oocyte development after sexual maturation, simultaneous maturation and atresia of oocytes occur before ovulation during the follicular phase of the menstrual cycle. This results in the generation of oocytes with different physiological patterns within a single ovary. Bulk sequencing can capture the global transcription profile of the samples, which cannot explain the differences between individual oocytes. The scRNA-seq technique can detect cell-to-cell heterogeneity and adequately identify the molecular mechanism involved in oocyte fate determination. Additionally, scRNA-seq can identify the individual components of the complex structures. 

Ovary comprises various types of cells, including some unidentified cells. The isolation of single cells from complex tissue or cellular structures comprising multiple cell types, such as follicles and ovarian vascular networks is a technical challenge. Additionally, oocytes form a small proportion of the entire ovarian cell population [65]. The isolation of cells with known markers using FACS can partially overcome this limitation. However, the global molecular profile of the entire tissue can be captured at the cellular level using scRNA-seq without the marker protein antibody-induced cell alteration.

Recently, Wagner et al. subjected the ovarian cortex, which is the reservoir of growing oocytes, to scRNA-seq analysis [65]. Their study aimed to examine the presence of oogonial stem cells (OSCs) in the adult ovary, which has been controversial. The DDX4-positive cluster was extracted, and the expression of OSC markers was examined. The expression levels of known OSC markers (DAZL, DPPA3, and PRDM113), germ cell/pluripotency markers (NANOG, POU5F1, and TFAP2C), or oocyte markers (GDF9, FIGLA, OOSP2, and ZP3) in DDX4-positive cells were higher than those in DDX4-negative somatic cells. Consistently, DDX4-positive cells were detected around the vasculatures in the ovarian cortex.

Furthermore, ovarian somatic cell populations exhibit cellular heterogeneity [66]. For example, granulosa cells (GCs) from small antral follicles with 1–2- mm diameter exhibited the EGR4^high^/FST^low^/VCAN^low^/WT1^high^ expression pattern. In contrast, GCs with 2–5-mm diameter that diverged into cumulus GCs exhibited the FST^high^, HTRA1^high^, IGFBP2^high^, IHH^high^, INHBB^high^, and VCAN^high^ expression pattern, while mural GCs exhibited the AKIRIN1^high^, CITED2^high^, EGR4^low^, KRT18^high^, LIHP^high^, and WT1^low^ expression pattern. Ovarian theca cells (TCs) also exhibited cellular heterogeneity. These TCs can be subcategorized into common progenitor TCs (pTCs), FOS-positive and JUN-positive stressed TCs, interna TCs (inTCs), and externa TCs (exTCs). Cell trajectory analysis revealed pseudotimes as follows: pTCs → stressed pTCs and pTCs → inTCs → exTCs → stressed exTCs or pTCs → stressed pTCs → stressed exTCs and pTCs → inTCs → exTCs.

## 8. Single-Cell-Based Study on Testicular Germ Cells

Various molecular events and epigenetic changes contribute to spermatogenesis, a complex process that leads to the production of mature sperm. The duration of spermatogenesis in mice and humans is approximately 35 and 42–76 days, respectively [67]. The interactions among germ cells, Sertoli cells, epithelial cells, and complete blood-testis-barrier are critical for spermatogenesis [68]. Spermatogenesis involves the following steps: proliferation and differentiation of spermatogenic stem cells (SSCs; spermatogonia), meiosis of spermatocytes, and maturation of spermatozoa [69]. The SSCs comprise 0.03% and 22% of total germ cells in mice and humans, respectively [70].

A spermatogonium (As) of mouse produces 16 undifferentiated clones through a series of mitotic divisions [As, Apr (pairs of spermatogonia, A_paired_), and Aal (4, 8, and 16 spermatogonia, A_aligned_)]. Additionally, spermatogonia undergo mitotic cycles (A1–4, intermediate, and B) to become large chains of differentiated cells and form the primary spermatocytes. Meanwhile, the spermatogonia of humans (A_dark/pale_) undergo four mitotic divisions to produce eight clones of stem cells. Thus, 40 million mature sperms are produced per gram of testis tissue despite the small pool of SSCs in mice (0.03%) [70,71]. Mouse spermatogonium cells are identified based on the expression of some critical genes, such as *Id4*, *Pax7*, *Bmi1*, *Eomes*, *Gfra1*, *Nanos2*, *Utf1*, *Plzf*, *Sall4*, and *Lin28*. Plzf, Sall4, and Lin28 are expressed in most spermatogonia [72,73,74,75], while Id4, Pax7, Bmi1, and Eomes are selectively expressed in mouse As [76,77,78,79]. Gfra1 and Nanos2 are expressed when As develops into Apr and Aal4 [80,81]. Additionally, several studies have revealed various markers of A_dark/pale_ spermatogonia in humans, including FGFR3, CD9, FMR1, GFRA1, GPR125, ID4, LIN28, PLZF, UCHL1, and UTF1 [82]. The marker of spermatogonia undergoing differentiation or differentiated spermatogonia in both humans and mice is cKIT [83,84,85]. Genome-wide and transcriptome studies are necessary to elucidate the molecular mechanism underlying spermatogenesis. 

Recent scRNA-seq studies have revealed that human spermatogonia can be subclassified into different groups based on gene expression profiles. Guo et al. classified spermatogonia into four states (states 1–4). State 1 spermatogonia are self-renewing quiescent adult SSCs that express stem cell-specific genes. The transition of state 1 cells to state 2 is mediated through the upregulation of the expression of DNA replication/repair factors and cell cycle-related genes as well as the downregulation of the expression of TXNIP (involved in glucose transport) and key stem cell-related TFs. Additionally, the downregulation of stem cell signaling, upregulation of RNA splicing, and enhancement of mitochondrial functions are critical for the transition of state 2 cells to state 3. For the transition to state 4, the expression of genes promoting spermatogonial differentiation must be upregulated [86].

Sohni et al. also identified four different subsets of human SSCs [87]. The authors named the subsets SSC-1, SSC-2, early differentiating spermatogonium (early diff-SPG), and differentiating SPG (diff-SPG). The expression of most known SSC markers, such as PIWIL4, LPPR3, CELF4, FSD1, EGR4, FGFR3, and TSPAN33, is upregulated in the SSC-1 subset. SSC-2 expresses some of the SSC markers and NANOS3. The cell cycle of most SSC-1 and SSC-2 subsets is arrested at the G0 or G1 phase, with some arrested at the S or G2-M phase. The early diff-SPG cells express ASB9, L1TD1, and NANOS3, whereas diff-SPG cells selectively express SOHLH2. Both early diff-SPG and Diff-SPG cells express CALR, DMRT1, DNMT1, and TUBA3D. Interestingly, the four transcriptionally distinct cell populations have also been observed in mouse and macaque [88,89,90]. The analysis of scRNA-seq data from the three species revealed six separate SPG populations (SPG1–6). The highest number of undifferentiated SPG cells was found in humans and macaques. The expression levels of CDK17, FMR1, MAGEB2, MORC1, MSL3, TCF3, TSPAN33, and ZBTB43 were highly upregulated in SPG1 cells. Additionally, PIWIL4 was identified as a common PGC1 marker for all three species. The mouse SPG3 cells share several markers with human and macaque SPG 3–5 cells, such as DMRT1, KIT, STRA8, SOHLH1, and SOHLH2. The SPG6 population may be a transition state between type B SPG and preleptotene spermatocytes as it expresses meiosis-related genes, such as *DNAJB11*, *LY6K*, *MEIOB*, *PRDM9*, *SPATA22*, *SYCP2/3*, *SYCE1/2*, and *TEX101*. Interestingly, A_dark_ and A_pale_ spermatogonia were transcriptionally indistinguishable and both cell populations were included in the SPG1 population. Similarly, Wang et al. also reported three different cell clusters based on scRNA-seq data [91]. 

## 9. Application of scRNA-seq for the Diagnosis of Reproductive Disorders

Recently, Ferrero et al. demonstrated that the scRNA-seq technique is a useful method to diagnose endometriosis and elucidate the mechanisms underlying its development [92]. The pathogenesis of most cases of endometriosis, which is characterized by the presence of endometrial tissue in the ovary and peritoneal cavity, is dependent on estrogen production [93]. The scRNA-seq of oocytes derived from patients with endometriosis and healthy donors revealed 520 DEGs (394 upregulated and 126 downregulated) between the two groups. Among the top 20 genes, *APOE*, *DUSP1*, *MGST1*, *G0S2*, *ID4*, *WEE1*, and *H2AFZ* expression levels were upregulated in the oocytes of patients with endometriosis. Functional enrichment analysis revealed that genes involved in mitochondrial function, steroid metabolism, response to oxidative stress, and cell growth regulation were markedly altered in the oocytes of patients with endometriosis.

Recently, Ye et al. compared the gene expression patterns between in vivo-matured and in vitro-matured metaphase II oocytes and identified approximately 500 DEGs [94]. Additionally, the expression of genes related to cell cycle, mRNA metabolism, and DNA metabolism has been found to be upregulated in in vivo-matured oocytes. In contrast, the expression of genes related to mitochondrial respiratory chain complex I biogenesis, response to endoplasmic reticulum stress, ATP metabolism has been found to be upregulated in the in vitro-matured oocytes.

## 10. Conclusions and Perspectives

The scRNA-seq is a powerful technology that is widely used in diverse research fields. In the future, scRNA-seq may have applications in reproductive medicine for molecular diagnosis, elucidating the mechanisms underlying reproductive failure, and novel drug development. Additionally, recent studies have developed advanced scRNA-seq methods to accurately quantify gene expression levels and enhance sensitivity. For example, Qiu et al. recently developed a new scRNA method called single-cell metabolically labeled new RNA tagging sequencing using 2,2,2-trifluoroethylamine/sodium periodate-based chemical reaction, which can be used to differentiate newly synthesized mRNAs from pre-existing mRNAs [95]. Further scRNA-seq studies are needed to improve the understanding of molecular mechanisms underlying human reproductive disorders and develop novel therapeutic strategies. Although studies have also demonstrated that the in vitro derivation of germ cells from stem cells necessarily mimics the processes of the in vivo germ cell development, more investigations are needed to improve the current methods so that the in vitro derived germ cells precisely represent in vivo germ cell differentiation. One of such efforts has been devoted to reconstituting three-dimensional (3D) gonad organoids [96,97]. Therefore, the organoid derived-germ cells could help to overcome the low germ cell numbers in nature and to explore function of the critical genes identified in the scRNA-seq analyses.

## Figures and Tables

**Table 1:** Summary of technical features of the scRNA-seq methods described in the review.

Methods	Summary	Advantages	Challenges
Smart-seq [10,11]	• 10^2^–10^3^ cells/run • Detects full-length transcript • Addition of a few cytosines on 5′ end of full-length transcript allows hybridization with oligonucleotide primer	• Available commercial kits • Detection of different splice variants	• No detection of strand-specific nature of mRNAs
CEL-seq [12,13]	• 10^2^–10^3^ cells/run • Only 3′-tag transcripts • Pipets single cell per tube	• Improved accuracy • Strand specificity and efficient barcoding	• Difficult to distinguish splice variants • Less sensitive
Qualtz-seq [14]	• 10^4^–10^5^ cells/run • Cell isolation using FACS • Barcoding cells and first round of PCR performed on individual cell	• High UMI conversion efficiency • Low cell/run cost	• High amplification error rate • Smaller fragments preference
MARS-seq [15]	• 10^3^–5 × 10^3^ cells/run • Cell isolation using FACS • Barcoding cells and first round of PCR performed on individual cell • Only 3′-tag transcripts	• Low reaction volume • Low noise • Strand specificity	• Not suitable for identifying splice variants • Limited to polyA RNAs • Requires FACS
Cyto-seq [16]	• 10^2^–10^4^ cells/run • Only 3′-tag transcripts • PCR amplification using gene-specific primers • Beads with unique barcodes used for barcoding and transcript amplification	• High throughput • No restriction on cell sizes	• Time-consuming • Trade-off between sequencing depth and detection of differential gene expression
SUPeR-seq [17]	• ~10 cells/run (micromanipulation) • Individual cell processing • Random primers with universal anchor sequence used for PCR amplification	• Detection of circular RNAs • 3′ bias avoidable	• Low throughput
Drop-seq [18]	• Split and pool synthesis of cell barcodes and UMI synthesis conducted on primer beads • cDNA amplification of transcripts of the cells carried within droplets • Only 3′-tag transcripts	• Low cost • Robust cell processing (10^4^ cells/day) • High yield • Customizable cell barcode	• High dependency on microfluidics
InDrop [19]	• Only 3′-tag transcripts • Polyacrylamide hydrogels with ssDNA primers with barcodes and polyT tails used • Each cell suspended in droplet with hydrogel and cell lysis proceeds within the droplet	• Low cell/run cost • Robust cell processing • High yield • Customizable cell barcode	• Low mRNA capture efficiency • One to one labeling of cell and barcode not guaranteed • High dependency on microfluidics
MATQ-seq [20]	• ~10^2^ cells/run • Cells mouth-pipetted into individual PCR tube • Barcodes incorporated to transcript from G enriched primers that bind to polyC tail	• Captures both polyA and non-polyA RNAs • Low 3′ end bias	• Low throughput
Chromium [21]	• 10^2^–10^4^ cells/run • Only 3′-tag transcripts • Barcoded gel beads, cells and enzymes partitioned by oil	• Robust cell processing • Automated procedures • Relatively high cell capture efficiency	• High dependency on microfluidics
sci-RNA-seq [22]	• Methanol fixation of cells • Only 3′-tag transcripts • Reverse transcription incorporates UMI and barcode to each cell • Transposase used prior to library amplification	• Minimized perturbance to cell state or RNA integrity • FACS step can be incorporated	• Low throughput
Seq-Well [23]	• Method largely follows Drop-seq method • Cells loaded into subnano liter well by gravity	• Microfluidics device-independent • Potential for multi omics measurement at single cell scale	• Not fully automated
DroNC-seq [24]	• Method largely follows Drop-seq method • Only 3′-tag transcripts • New microfluidics design and nuclei isolation incorporated to the original Drop-seq method	• Reduced nuclei isolation time • Minimized RNA degradation	• High dependency on microfluidics
SPLiT-seq [25]	• ~5 × 10^4^ cells/run • Cell or nuclei are fixed with formaldehyde • Only 3′-tag transcripts • Transcriptome identification performed by four rounds of combinatorial barcoding • Barcoded samples undergo PCR amplification and are pooled to be sequenced	• Minimized perturbance to cell state or RNA integrity • Independent of microfluidics device	• Low number of average read/cell • Low cell type differentiation resolution

## Abbreviations

DEG	Differentially expressed gene
ESC	Embryonic stem cell
FACS	Fluorescence-activated cell sorting
GC	Granulosa cells
hPGC	Human primordial germ cell
MACS	Magnetic-activated cell sorting
OSC	Oogonial stem cell
PCG	Primordial germ cell
pre-PGC	Progenitor primordial germ cell
RA	Retinoic acid
scRNA-seq	Single-cell RNA sequencing
SC-seq	Single-cell sequencing
SSC	Spermatogenic stem cells
TC	Theca cell
TF	Transcription factors
t-SNE	t-stochastic neighbor embedding
UMI	Unique molecular identifier
